# *Ascophyllum nodosum* Extract and Mycorrhizal Colonization Synergistically Trigger Immune Responses in Pea Plants against Rhizoctonia Root Rot, and Enhance Plant Growth and Productivity

**DOI:** 10.3390/jof8030268

**Published:** 2022-03-07

**Authors:** Younes M. Rashad, Hany H. A. El-Sharkawy, Nahla T. Elazab

**Affiliations:** 1 Plant Protection and Biomolecular Diagnosis Department, Arid Lands Cultivation Research Institute, City of Scientific Research and Technological Applications (SRTA-City), New Borg El-Arab City 21934, Egypt; 2 Agricultural Research Center, Department of Mycology Research and Plant Disease Survey, Plant Pathology Research Institute, Giza 12211, Egypt; drhanylasheen@yahoo.com; 3 Botany Department, Faculty of Science, Mansoura University, Mansoura 35516, Egypt; nahlatharwat83@gmail.com

**Keywords:** *Ascophyllum nodosum*, mycorrhizal fungi, pea, *Rhizoctonia solani*, root rot, seaweed

## Abstract

Rhizoctonia root rot is one of the most destructive diseases affecting pea crops, resulting in up to 75% loss. In this study, the biocontrol activity of seaweed (*Ascophyllum nodosum*) extract at 1, 2, and 3% and/or mycorrhization of pea roots was investigated against Rhizoctonia root rot under greenhouse conditions. In addition, their effects on the transcriptional, physiological, ultrastructural, and growth status of pea plants were also studied. The results showed that the mycorrhizal colonization of pea roots and the application of the seaweed extract at 3% synergistically overexpressed the responsive factor (*JERF3*) recording 18.2-fold, and the defense-related genes peroxidase (23.2-fold) and chitinase II (31.8-fold). In addition, this treatment improved the activity of the antioxidant enzymes POD and PPO, increased the phenolic content in pea roots, and triggered multiple hypersensitivity reactions at the ultrastructural level of the cell, leading to a 73.1% reduction in disease severity. Moreover, a synergistic growth-promoting effect on pea plants was also observed. The photosynthetic pigments in pea leaves were enhanced in response to this dual treatment, which significantly improved their yield (24 g/plant). The inducing effect of mycorrhizal colonization on plant resistance and growth has been extensively studied. However, developing improved and synergistically acting biological agents for plant disease control and growth promotion as alternatives to the chemical fungicides is crucial for safety and food security. Based on these results, it can be concluded that the mycorrhizal colonization of pea roots and soaking their seeds in the *A. nodosum* extract at 3% have a promising and improved biocontrol activity against *R. solani*, and a growth-promoting effect on pea plants. However, field applications should be evaluated prior to any use recommendations.

## 1. Introduction

Pea (*Pisum sativum* L.), a herbaceous legume that belongs to the family Fabaceae, is widely grown all over the world, mainly in the cold climate areas, as it can withstand freezing temperatures down to −13 °C [1]. It has a rich nutritional value with high amounts of protein, carbohydrates, vitamins, minerals, and fibers. This makes it a valuable food commodity for human consumption and animal feed [2]. In 2020, the world cultivated area under green peas, which was estimated at around 2.5 million ha, produced 19.8 million tones [3]. However, pea is subjected to various fungal diseases, which significantly reduce both yield and quality of pea.

Rhizoctonia root rot, caused by *Rhizoctonia solani* Kühn, is one of the most destructive diseases affecting pea, especially in warm and moist conditions. The causal pathogen can attack a wide range of hosts, mostly at any age, and under a diverse array of soil conditions of temperatures, textures, pH, and moisture [4,5]. Disease symptoms include seed rotting, seedling damping-off, root and stem rotting, reddish-brown lesions on hypocotyl and epicotyl regions, yellowing of leaves, stunting, and death [6]. Isolates belonging to different anastomosis groups (AGs) of *R. solani* can cause pea root rot [7]. However, *R. solani* AG 4 is typically the most virulent one in this concern, causing up to a 75% reduction in the yield [8]. Various chemical fungicides have been reported to control this pathogen, such as hexaconazole, difenoconazole, carbendazim, and mancozeb [9]. However, disease control using chemical fungicides may be undesirable, owing to their adverse effects on human and animal health, the diversity and composition of soil microbial communities, and environment [10]. Biological control represents an eco-friendly alternative to the chemical fungicides and a promising strategy for plant diseases management [11].

Arbuscular mycorrhizal fungi (AMF) are obligate biotrophs (subphylum Glomeromycotina) that live in a mutualistic relationship with roots of the majority of terrestrial plants [12]. Multiple benefits are received by the plant partner due to association with AMF, such as improving the plant growth [13], enhancing nutrients uptake, inducing plant tolerance to salinity and drought stresses [14], and triggering plant resistance against different plant diseases [15]. The application of AMF as a biocontrol agent against different plant diseases including Rhizoctonia root rot has been widely studied by many researchers all over the world [16,17,18,19]. In this regard, Abdel-Fattah et al. [20] reported a considerable reduction in the disease severity in mycorrhizal common bean plants infected with Rhizoctonia root rot, when compared with the non-mycorrhizal ones.

Seaweeds are macroalgae from different algal categories, including brown, red, and green algae, which may be utilized as a food by some coastal communities [21], animal feed, a source of bioactive compounds for industrial applications such as alginate, agar, and carrageenan [22], and the production of biofuels. The growth promoting effect of some seaweed extracts on different plant species has also been reported [23]. Furthermore, the antifungal activity of some seaweed extracts against many phytopathogenic fungi has been reported [24]. In this concern, Graff and Raj [25] reported the biocontrol activity of *Sargassum tenerrimum* extract at 20% against the sheath blight of rice caused by *R. solani*. The biocontrol activity was attributed to the antifungal compound n-hexadecanoic.

Plants tend to activate multiple defense responses in order to defend themselves against pathogen attack. One of the main defense responses is triggering various signaling pathways such as salicylic acid, jasmonic acid, and ethylene pathways that regulate many defense-related genes in the plant [14]. Crosstalks between these signaling pathways may form the net plant resistance to the pathogen. Jasmonate and ethylene-responsive factor 3 (*JERF3*) is a member of the *ERF* transcription factor family which regulates a cascade of defense-related genes through both ethylene and jasmonate signaling pathways against different biotic and abiotic stresses [26]. One of the defense-related genes regulated via the jasmonic acid pathway is the chitinase encoding gene (*CHI*), which encodes the chitinase protein, a crucial antifungal enzyme against different fungal pathogens. It catalyzes the hydrolysis of the chitin molecules, the main component of the fungal cell walls [27]. The peroxidase gene (*POD*) encodes the antioxidant protein, which is involved in oxidizing the phenolic substances scavenging reactive oxygen species produced by different stresses [28]. The overexpression of these genes leads to the induction of plant immunity against pathogenic fungi. This study aimed to study (1) the biocontrol activity of seaweed application and colonization of pea roots with AMF against Rhizoctonia root rot under greenhouse conditions, (2) effects of these treatments on the expression profile of the responsive factor *JERF3* and two defense-related genes, (3) effects on the ultrastructure of pea root, and (4) impacts on the growth and antioxidant status of pea plants.

## 2. Materials and Methods

### 2.1. Pea Cultivar, Seaweed, and Fungal Inocula

Pea seeds of Master-B cultivar, obtained from Horticultural Research Institute, Agricultural Research Center, Giza, Egypt, were used in the greenhouse experiment. Seaweed extract (*Ascophyllum nodosum* L.) used in this study was purchased from Qingdao Blue Treasure Seaweed Biotech Co., Ltd. (Qingdao, China). Analysis of the seaweed extract is presented in Table 1.

A highly virulent isolate of *R. solani* (AG-2-2 IIIB), originally isolated from rotted roots of common bean plant, was kindly provided by Plant Pathology Research Institute, Agricultural Research Centre, Egypt. For inoculum preparation, flasks containing sterilized soil: sorghum (2:1 *v*/*v*) medium was inoculated with discs from a 5-day-old culture of the pathogen, and incubated at 26 ± 2 °C for two weeks.

A mixed inoculum of AMF (78% colonization index), kindly obtained from Plant Pathology Research Institute, Agricultural Research Centre, Egypt, was used in this study. The inoculum was composed of spores of *Rhizophagus irregularis* (Blaszk., Wubet, Renker and Buscot) Walker and Schüßler, *Funneliformis mosseae* (Nicolson and Gerd.) Walker and Schüßler, *Rhizoglomus clarum* (Nicolson and Schenck) Sieverd., Silva and Oehl, *Gigaspora margarita* (Becker and Hall), and *G. gigantea* (Nicol. and Gerd.) Gerd. and Trappe, in equal ratio.

### 2.2. Screening for Antifungal Activity of the Seaweed Extract

The seaweed extract was screened in vitro for its antifungal activity against *R. solani* using the agar plate technique. An aqueous extract of the seaweed was added to sterilized potato dextrose agar (PDA) plates before solidification to prepare 1, 2, and 3% final concentrations. PDA plates supplemented with a synthetic fungicide (nystatin) at 50 µg/mL were used as a positive control. Another set of PDA plates treated with sterile water was used as a negative control. All plates were singly inoculated with discs from a 5-day-old culture of *R. solani*, and incubated at 26 ± 2 °C for 6 days. Inhibition in the fungal growth was determined. Three replicates were used for each treatment.

### 2.3. Greenhouse Experiment

The greenhouse experiment was conducted at Tag El-Ezz Agricultural Research Station, Agricultural Research Center, Egypt. Plastic pots (30 cm diameter) filled with sterilized soil (clay: sand, 2:1, *v*/*v*) were used in this experiment. At growing time, half of the used pots were inoculated with an AMF inoculum as a seed bed (10 g seed^−1^). Pea seeds were surface-sterilized using 1% sodium hypochlorite solution, soaked in sterilized water for 3 h, and ten seeds were planted per pot. For the seaweed application, pea seeds were soaked before planting for 3 h in a seaweed aqueous extract at 1, 2, and 3% amended with gum arabic (1%). For chemical fungicide application, pea seeds were soaked before planting for 3 h in a solution of the chemical fungicide Tendro 40% FS (Carboxin 20% + Thiram 20%) at the recommended dose (3.5 mL/kg seeds). Thirty days post-planting, the soil was infested by mixing the pathogen inoculum thoroughly with the upper layer of the soil at 3% (*w*/*w*). Pots treated only with water were used as a negative control. For each treatment, ten pots were used. All pots were regularly irrigated and no fertilizers were applied. Pots were arranged in a completely randomized design under greenhouse conditions. The applied treatments in this experiment were as follows: C: uninfected and non-mycorrhizal, CM: uninfected and mycorrhizal, P: infected and non-mycorrhizal, PM: infected and mycorrhizal, PF: infected, non-mycorrhizal and treated with fungicide, PFM: infected, mycorrhizal, and treated with fungicide, SW1: uninfected, non-mycorrhizal, and treated with the seaweed extract at 1%, SW2: uninfected, non-mycorrhizal, and treated with the seaweed extract at 2%, SW3: uninfected, non-mycorrhizal, and treated with the seaweed extract at 3%, SW1M: uninfected, mycorrhizal, and treated with the seaweed extract at 1%, SW2M: uninfected, mycorrhizal, and treated with the seaweed extract at 2%, SW3M: uninfected, mycorrhizal, and treated with the seaweed extract at 3%, PSW1: infected, non-mycorrhizal, and treated with the seaweed extract at 1%, PSW2: infected, non-mycorrhizal, and treated with the seaweed extract at 2%, PSW3: infected, non-mycorrhizal, and treated with the seaweed extract at 3%, PSW1M: infected, mycorrhizal, and treated with the seaweed extract at 1%, PSW2M: infected, mycorrhizal, and treated with the seaweed extract at 2%, and PSW3M: infected, mycorrhizal, and treated with the seaweed extract at 3%. All pots were kept under greenhouse conditions (27/16 °C (day/night), 10 h light period and 62% relative humidity).

#### 2.3.1. Expression Profiles of Defense-Related Genes

Seven days post-inoculation (dpi), pea roots from each treatment were sampled for molecular investigation. mRNA from pea roots was extracted using RNeasy Mini Kit (Qiagen, Hilden, Germany) based on the manufacturer’s instructions. For cDNA synthesis, a reaction mixture (20 μL) was used by mixing RNase free water (3.8 μL), 5× reaction buffer (3 μL), oligo (dT) primer (5 pmol μL^−1^, 7 μL), dNTPs (10 mM, 3 μL), RNA (30 ng, 3 μL) and reverse transcriptase enzyme (New England Biolabs, Germany) (0.2 μL). The reverse transcription reaction was carried out using a SureCycler 8800 (Agilent, Santa Clara, CA, USA) at 42 °C for 1 h, and then at 70 °C for 10 min, the product was stored at −80 °C.

The quantitative Real-Time PCR (qPCR) reaction (20 μL) was composed of cDNA (3 μL), 2xSYBR^®^ Green RT Mix (Bioloine, Hirrlingen, Germany) (12.5 μL), forward and reverse primers (10 pmol μL^−1^) (1.5 μL for each), and RNase free water (1.5 μL). Primer sequences of the three tested genes are presented in Table 2. The qPCR program was as follows: one cycle at 95 °C for 3 min, 45 cycles (95 °C for 15 s, 56 °C for 30 s and 72 °C for 30 s) using a Rotor-Gene-6000-system (Qiagen, Santa Clarita, CA, USA). Elongation factor 1 α (EF1 α) was used as a reference gene for its high stability in arbuscular mycorrhizal plants [29]. The relative expression was calculated using the comparative CT method (2^−∆∆CT^) [30]. For each sample, triplicate biological and technical replications were used.

#### 2.3.2. Growth and Yield Parameters

Twenty dpi, ten random plants for each treatment, were carefully uprooted, washed under running water, and evaluated for shoot height (cm), root length (cm), shoot and root dry weight (g), the number of leaves per plant, and leaf area (cm^2^). Forty dpi, ten random plants for each treatment, were evaluated for yield and its components [number of green pods per plant, weight of pod (g), length and width of pod (cm), number of seeds per pod, and yield per plant (g)]. All weights were determined after the samples drying in a hot air oven (80 °C) until a constant weight.

#### 2.3.3. Disease Assessment

Twenty dpi, ten plants from each treatment, were evaluated for the disease severity (DS) of Rhizoctonia root rot using 5-degrees-scale according to Carling et al. [31], where 0 = no damage, 1 = minor discoloration of hypocotyl, 2 = discoloration plus small necrotic lesions on hypocotyl, 3 = discoloration with large necrotic lesions on hypocotyl, and 4 = full death. The following equation was used to calculate DS:

$$\mathrm{DS}\;\%=\frac{\Sigma ab}{AK}\times 100$$

where *a* = number of infected plants at the same severity grade, *b* = severity grade, *A* = total number of evaluated plants, and *K* = the highest severity grade.

#### 2.3.4. Phenolic Content and Activity of Antioxidant Enzymes

Twenty dpi, the phenolic content (PC), and the activity of the antioxidant enzymes peroxidase (POD) and polyphenol oxidase (PPO) were determined in the pea roots of each treatment. PC was estimated in pea roots according to Malick and Singh [32]. One gram of pea root was ground in ethyl alcohol (80%, 10 mL), and centrifuged at 4000 rpm for 20 min. The supernatant was collected, evaporated to dryness, then 5 mL distilled water was added. In a clean test tube, 0.2 mL of the solution was added and made up to 3 mL with distilled water, to which 0.5 mL of Folin–Ciocalteu reagent was added. After 3 min, 2 mL of 20% Na_2_CO_3_ solution was added. The contents were mixed thoroughly and placed in a boiling water bath for 1 min, cooled, and the absorbance was measured at 650 nm against blank. For each treatment, three replicates were used.

For the preparation of the enzyme crude extract, 1 g of the plant root was ground with 2 mL potassium phosphate buffer (0.2 M, pH 7.0) and centrifuged at 4000 rpm for 20 min at −4 °C. The activity of POD and PPO enzymes was estimated according to Maxwell and Bateman [33] and Galeazzi et al. [34], respectively. For each treatment, three replicates were used.

#### 2.3.5. Total Photosynthetic Pigments and Biochemical Analyses

Twenty dpi, total photosynthetic pigments in pea leaves, were estimated as described by Harborne [35]. In thirty dpi, the electrolyte leakages in plant roots were assessed to determine the membrane permeability (MP%) according to Shi et al. [36], using an EC Meter (Hana Instruments, Bedfordshire, UK). Total soluble solids (TSS, Brix) were determined in the fresh pea seeds using a hand refractometer model Master T (ATAGO Co., Ltd., Tokyo, Japan). For each treatment, three replicates were used.

#### 2.3.6. Estimation of Mycorrhizal Colonization

Thirty dpi, three pea plants from each treatment, were carefully uprooted, washed with tap water, and their roots were evaluated for mycorrhizal colonization. The roots were cut into segments (1 cm), stained with 0.05% trypan blue (Sigma, St. Louis, MO, USA) according to Phillips and Hayman [37], and microscopically examined for the estimation of their mycorrhizal colonization level according to Trouvelot et al. [38].

#### 2.3.7. Ultrastructural Investigation

In fifteen dpi, pea roots were sampled and processed for a transmission electron microscopy (TEM) observation. A section (1–2 cm^2^) of plant root was dehydrated using serial concentrations of ethanol up to 100%, each stage for 10 min. The specimen was then embedded in gelatin capsules filled with fresh Araldite, and heated in an oven at 60 °C for 60 h. Using Reichert Ultramicrotome, ultrathin sections were obtained. The sections were picked up on a 200-mesh copper grid, and stained with uranyl acetate, followed by lead citrate, and examined using a transmission electron microscope (JEM-1230; JEOL Ltd., Tokyo, Japan).

### 2.4. Statistical Analyses

Data obtained in this study were analyzed using the software CoStat (version 6.4). The data were first examined for normality and then subjected to analysis of variance. Comparisons between the means were performed using Tukey’s HSD test at *p* ≤ 0.05 based on one-way ANOVA.

## 3. Results

### 3.1. Expression Profiles of Defense-Related Genes

**Figure 1:**
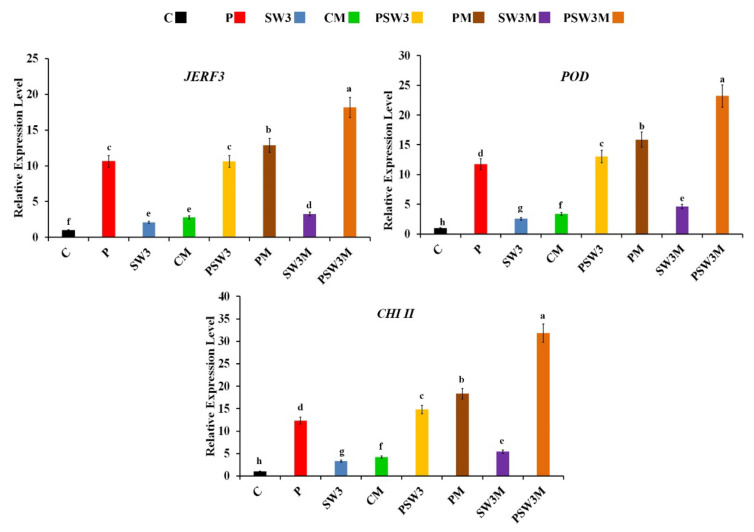
Histograms showing the relative transcriptional expression levels of the jasmonate and ethylene-responsive factor 3 (*JERF3*) and two defense-related genes; peroxidase (*POD*) and chitinase II (*CHI II*) in roots of pea infected with Rhizoctonia root rot in response to treating with the seaweed extract at 3% and/or colonization with arbuscular mycorrhizal fungi, seven days post-pathogen inoculation. Where, C: non-treated control, CM: uninfected and mycorrhizal, P: infected and non-mycorrhizal, PM: infected and mycorrhizal, SW3: uninfected, non-mycorrhizal, and treated with the seaweed extract at 3%, SW3M: uninfected, mycorrhizal, and treated with the seaweed extract at 3%, PSW3: infected, non-mycorrhizal, and treated with the seaweed extract at 3%, and PSW3M: infected, mycorrhizal, and treated with the seaweed extract at 3%. For each gene, columns superscripted with the same letter are not significantly different according to Tukey’s HSD test at *p* ≤ 0.05. Each value represents the mean of three biological replicates; each sample was analyzed in triplicate. Error bars represent standard errors.

Expression profiles of the jasmonate and ethylene-responsive factor 3 (*JERF3*) and two defense-related genes, namely, peroxidase (*POD*) and chitinase class II (*CHI II*) in pea roots at 7 dpi, in response to treating with the seaweed extract and/or colonization with AMF, were determined using qPCR (Figure 1).

For *JERF3*, the results showed that all applied treatments provoked the gene expression, at varying extents, compared with the untreated nonmycorrhizal control. In this regard, the colonization of the infected roots with AMF significantly upregulated the gene expression higher than the other single or dual treatments, when compared with the untreated nonmycorrhizal treatment. The highest expression was recorded for the infected pea roots, which were treated with the seaweed extract at 3%, and colonized with AMF (18.2 fold). Regarding *POD*, the data obtained revealed that all tested treatments considerably triggered the gene expression, at varying degrees, compared with the untreated nonmycorrhizal treatment. In this concern, the mycorrhizal-infected roots showed gene expression higher than the infected-nonmycorrhizal roots and the infected-nonmycorrhizal roots which were treated with the seaweed extract at 3%. However, the highest expression was observed for the infected mycorrhizal roots, which were treated with the seaweed extract at 3% (23.2-fold). For *CHI II*, the applied single, dual, or triple treatments differentially induced gene expression. The obtained results showed that the mycorrhization of the infected roots led to a gene upregulation higher than that recorded for the other single or dual treatments, compared with the untreated nonmycorrhizal treatment. The maximum gene expression was recorded for the infected mycorrhizal roots, which were treated with the seaweed extract at 3% (31.8 fold).

### 3.2. Effect on the Growth and Yield Parameters

The mean growth parameters of pea plants infected with Rhizoctonia root rot in response to treatment with the seaweed extract and/or colonization with AMF at 20 dpi are presented in Table 3. The results obtained showed that treating pea plants with the seaweed extract at 2 and 3% enhanced their shoot height, root length, shoot and root dry weight, and leaf area, but did not affect the number of leaves per plant, compared with the untreated-nonmycorrhizal plants. Meanwhile, the colonization of pea roots with AMF enhanced all evaluated growth parameters. The dual treatments with the seaweed extract at 2 or 3% and colonization with AMF were more effective than the single treatments. In this regard, the highest values of the evaluated parameters were recorded for the mycorrhizal plants treated with the seaweed extract at 3%.

In contrast, infection with Rhizoctonia root rot led to significant reduction in all evaluated growth parameters, except the number of leaves per plant, when compared with the untreated nonmycorrhizal plants. The mycorrhization of the infected plants considerably enhanced all evaluated growth parameters, compared with the infected nonmycorrhizal plants. This enhancing effect was significantly higher than that obtained, due to treating the infected plants with the chemical fungicide, except for the leaf area. In most of the evaluated growth parameters, treatment of the infected mycorrhizal plants with the chemical fungicide showed an enhancing effect less than that of the infected mycorrhizal plants which did not receive the chemical fungicide. Except for the number of leaves per plant, treatment with the seaweed extract at 2 or 3% mitigated the adverse effects of the infection on the evaluated parameters, compared with the untreated–infected plants. Mostly, no difference was observed between the dual treatment of the infected mycorrhizal plants treated with the seaweed extract at 3%, and the single treatments.

The data presented in Table 4 show the mean yield and its components of pea plants infected with Rhizoctonia root rot, in response to treatment with the seaweed extract and/or colonization with AMF at 40 dpi. Results from the greenhouse experiment revealed that treating pea plants with the seaweed extract at 3% considerably improved the yield, number of green pods per plant, pod weight, and pod length, but did not affect the pod width or number of seeds per pod, compared with the untreated nonmycorrhizal plants. In addition, the mycorrhization of the pea plants also showed an enhancing effect on the yield, the number of pods per plant, and pod weight, but not on the pod length or width, and the number of seeds per pod, when compared with the untreated nonmycorrhizal plants. However, the dual treatments of mycorrhizal plants with the seaweed extract were more effective than the single treatments. In this regard, the highest values were recorded for the mycorrhizal plants, which were treated with the seaweed extract at 3%, compared with the untreated nonmycorrhizal plants.

In contrast, the infection of pea plants with *R. solani* significantly reduced the evaluated parameters, except the pod length and number of seeds per plant, compared with the uninfected nonmycorrhizal plants. The colonization of infected pea plants with AMF mostly mitigated the adverse effect due to infection, compared with the infected nonmycorrhizal plants. Treatment of the infected plants with the seaweed extract at any concentration also mitigated the adverse effects in all parameters due to infection, compared with untreated nonmycorrhizal-infected plants. However, no significant difference was observed between the dual treatments (the seaweed extract and AMF) and the single ones regarding the evaluated yield parameters.

### 3.3. Effect on Disease Severity

**Figure 2:**
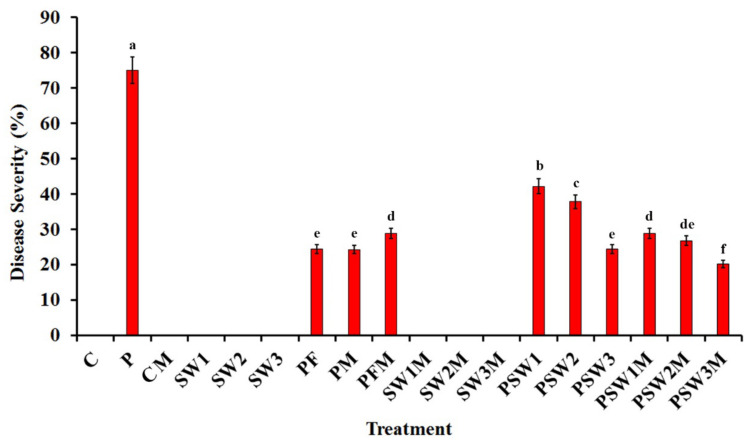
Histogram showing the disease severity in pea plants (cv. Master-B) infected with Rhizoctonia root rot in response to treating with seaweed extract and/or colonization with arbuscular mycorrhizal fungi at 20 days post inoculation. Where, C: uninfected and non-mycorrhizal, CM: uninfected and mycorrhizal, P: infected and non-mycorrhizal, PM: infected and mycorrhizal, PF: infected, non-mycorrhizal and treated with fungicide (Tendro 40% FS), PFM: infected, mycorrhizal, and treated with fungicide, SW1, SW2, and SW3: uninfected, non-mycorrhizal, and treated with the seaweed extract at 1, 2, and 3%, respectively, SW1M, SW2M, and SW3M: uninfected, mycorrhizal, and treated with the seaweed extract at 1, 2, and 3%, respectively, PSW1, PSW2, and PSW3: infected, non-mycorrhizal, and treated with the seaweed extract at 1, 2, and 3%, respectively, PSW1M. PSW2M, and PSW3M: infected, mycorrhizal, and treated with the seaweed extract at 1, 2, and 3%, respectively. Columns superscripted with the same letter are not significantly different according to Tukey’s HSD test at *p* ≤ 0.05. Each value represents the mean of ten replicates. Error bars represent standard errors.

The disease severity of pea plants infected with Rhizoctonia root rot in response to treatment with the seaweed extract and/or colonization with AMF at 20 dpi is illustrated in Figure 2. No disease symptoms were observed in pea plants, which were not infected with *R. solani*. The highest disease severity was recorded for the infected nonmycorrhizal pea plants, which were not treated with the seaweed extract, recording 75%. The obtained results indicated that the colonization of the infected plants with AMF and/or treatment with seaweed extract at any concentration significantly reduced disease severity, compared with the untreated nonmycorrhizal-infected plants. No significant difference was observed between disease severities of the infected pea plants, which were colonized with AMF or treated with the seaweed extract at 3%, and were treated with the chemical fungicide. However, the dual treatments of infected plants showed a disease severity lower than that of the single ones. In this regard, the lowest disease severity was recorded for the infected plants, which were colonized with AMF and treated with the seaweed extract at 3%, recording 20.2%.

### 3.4. Effect on Phenolic Content, Activity of POD and PPO Enzymes, Electrolyte Leakage, and TSS

Table 5 represents the mean phenolic content, activity of POD and PPO enzymes, electrolyte leakage, and TSS in pea roots infected with Rhizoctonia root rot in response to treatment with seaweed extract and/or colonization with AMF at 20 dpi. The obtained results indicated that mycorrhizal colonization with AMF and/or treating with the seaweed extract significantly induced the enzyme activity of POD and PPO, and enhanced the phenolic content and TSS in pea roots, compared with the untreated nonmycorrhizal plants. Meanwhile, no significant difference was observed for these treatments with regard to the electrolyte leakage, compared with the untreated-nonmycorrhizal plants. In addition, infection of pea roots with Rhizoctonia root rot led to a considerable increase in the phenolic content, the activity of POD and PPO enzymes, and electrolyte leakage, as well as a decrease in the TSS, compared with the untreated, uninfected nonmycorrhizal plants. The mycorrhization of the infected roots and/or treating with the seaweed extract significantly triggered the phenolic content, activity of POD and PPO enzymes, and TSS, and reduced the electrolyte leakage.

However, the dual treatments were more effective than the single ones in this regard. The highest values of phenolic content, and the activity of POD and PPO enzymes, were recorded for the infected mycorrhizal pea plants treated with the seaweed extract at 3%.

### 3.5. Effect on Total Photosynthetic Pigments

Data on the application of the seaweed extract and/or colonization with AMF on total photosynthetic pigments in the leaves of pea plants infected with Rhizoctonia root rot at 20 dpi are presented in Table 6. The obtained results showed that the mycorrhization of the pea plants and/or treatment with the seaweed extract at 3% significantly improved the total photosynthetic pigments, compared with the untreated nonmycorrhizal plants. However, treatment with the seaweed extract at 3% was more enhanced than the mycorrhization effect, and the dual treatment had more of an enhancing effect than the single treatments. In this regard, the highest content of the total photosynthetic pigments was recorded for the pea plants treated with the seaweed extract at 3% and/or colonized with AMF.

In contrast, the infection of pea plants considerably reduced the total photosynthetic pigments, when compared with the uninfected nonmycorrhizal plants. The mycorrhization of the infected pea plant and/or their treatment with the seaweed extract at any concentration significantly mitigated the adverse effect of the infection of the total photosynthetic pigments. However, the dual treatments were more effective than the single ones in this regard.

### 3.6. Effect on Mycorrhizal Colonization

**Figure 3:**
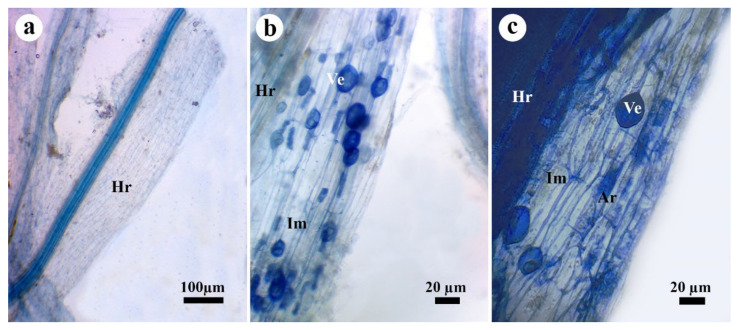
Pea root showing typical mycorrhizal structures. Where, (**a**) nonmycorrhizal root, (**b**) and (**c**) mycorrhizal roots, Hr = host root, Im = interaradical mycelia, Ve = vesicle, and Ar = arbuscule.

Mycorrhizal colonization in the roots of pea infected with Rhizoctonia root rot and/or treated with the seaweed extract at 30 dpi is presented in Table 7. The data obtained revealed that no mycorrhization was detected in pea plants that did not receive the AMF inoculum. On the contrary, the other treatments treated with AMF inoculum showed mycorrhizal colonization at varying degrees, when comped with the untreated nonmycorrhizal plants. Different typical mycorrhizal structures were observed in the pea roots via the microscopical examination (Figure 3). The obtained results indicated that the uninfected pea plants treated only with AMF inoculum had the highest degrees of colonization frequency (88.3%), colonization intensity (50.7%), and the frequency of arbuscules (21.6%).

The infection of pea roots with *R. solani* significantly reduced their mycorrhization level. Moreover, infected mycorrhizal pea plants treated with chemical fungicide showed a mycorrhization level lower than that not treated with the fungicide. The treatment of pea plants with the seaweed extract at any concentration did not affect their frequency of colonization or arbuscules, but reduced their colonization intensity at 2 and 3%. The treatment of the infected mycorrhizal pea plants with the seaweed extract at any concentration mostly did not affect their colonization level.

### 3.7. TEM Observations

**Figure 4:**
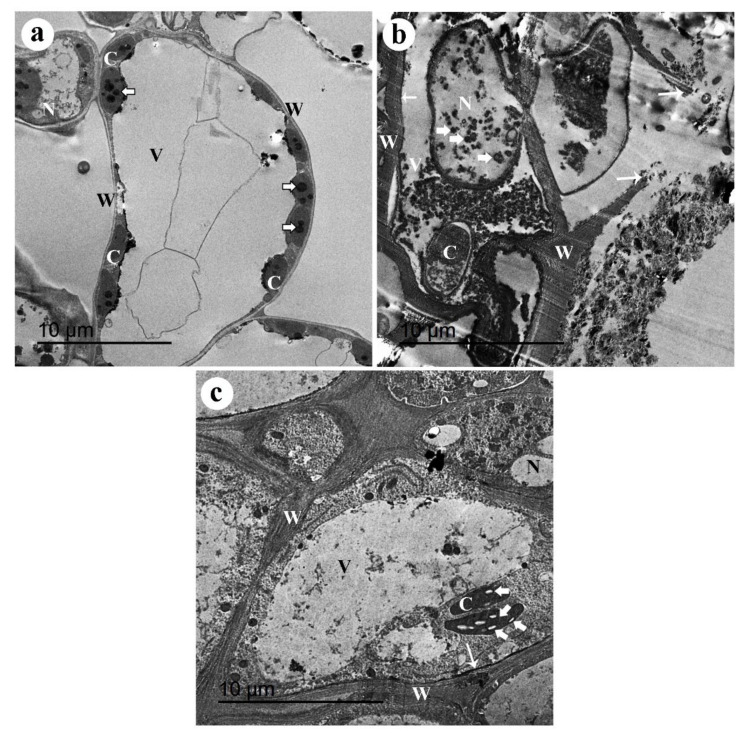
Transmission electron micrographs of pea roots infected with *R. solani*, colonized with arbuscular mycorrhizal fungi, and treated with the *A. nodosum* extract at 3%. Where (**a**) uninfected-untreated-nonmycorrhizal pea root showing normal cortical cell enclosed by a thin cell wall (W) and containing big vacuole (V), and a number of chloroplasts, with electron-dense plastoglobuli (arrowheads), (**b**) infected-untreated-nonmycorrhizal pea root showing a disorganized cell enclosed with a thick cell wall (W) and cell membrane (short arrow), abnormal chloroplast (C), big nucleus (N) containing electron-dense bodies (arrowheads), and a big vacuole (V). Note the disintegrated cell wall (long arrow) and the cytoplasmic components leaked out of the raptured cell, and (**c**) mycorrhizal pea root which infected with *R. solani* and treated with the *A. nodosum* extract at 3% showing well-organized cell with thick cell wall (W) and cell membrane (short arrow), big chloroplasts (C) containing starch granules (wide arrows), granulated cytoplasm, big vacuole (V) and normal nucleus (N).

Observations from the TEM of the uninfected untreated nonmycorrhizal pea root showed normal cortical cells enclosed within thin cell walls and cell membranes, and containing big vacuoles, small nuclei without nucleoli, and a number of chloroplasts that contain electron-dense plastoglobuli. Because these cortical cells were taken from the upper green part of the hypocotyl, some chloroplasts were observed in the examined cells (Figure 4a). The TEM examination of cortical cells from the infected, untreated nonmycorrhizal pea root revealed many ultrastructural alterations in response to the infection with *R. solani*. These ultrastructural alterations included disorganized cells enclosed in disintegrated thick cell walls and cell membranes, cytoplasmic granulation, electron-dense bodies, abnormal chloroplasts, and big nuclei and vacuoles. Moreover, the cytoplasmic components leaked out of the raptured cells (Figure 4b). The TEM observations of the mycorrhizal pea root infected with *R. solani*, and treated with the A. nodosum extract at 3%, showed well-organized cells with thick cell walls and cell membranes, and big chloroplasts containing starch granules, granulated cytoplasm, big vacuoles, and normal nuclei (Figure 4c).

## 4. Discussion

Rhizoctonia root rot is considered one of the most destructive diseases affecting pea, resulting in an economic loss of up to 75%. Moreover, the host range of the causal agent extends to a wide range of hosts at any age, and with different conditions. In this study, the biocontrol activity of the seaweed extract and/or the mycorrhization of pea roots was investigated against Rhizoctonia root rot with greenhouse conditions. In addition, they affected the transcriptional expression level of defense-related genes.

The obtained results revealed the synergistic biocontrol activity of the dual treatment in reducing the disease severity. The application of *A. nodosum*-based products in the disease management of different plant diseases has been investigated by many researchers [39]. In this regard, Abkhoo and Sabbagh [40] reported a significant reduction in the severity of the Phytophthora damping-off of cucumber, caused by *P. melonis*, when treated with Marmarine (a seaweed extract from *A. nodosum*) at 1%, as a root drench. This treatment was found to enhance plant resistance responses. Activities of different defense-related enzymes were induced, and the expression levels of many defense-related genes were also triggered due to this treatment. The obtained results in our study are in agreement with these findings. The induction of pea resistance against Rhizoctonia root rot due to the application of the *A. nodosum* extract at 3% is one of the most important findings in this study. The obtained results showed the upregulating effect of this treatment on the studied defense-related genes. Jasmonate and ethylene-responsive factor 3 (*JERF3*) is a transcription factor which controls the expression of a group of defense-related genes via both jasmonate and ethylene pathways in the plant, in response to different stresses [14]. The triggered expression of *JERF3*, which was reported in this study, may discuss the resistance-inducing effect against Rhizoctonia root rot in pea. To this end, an upregulation in the transcript level of the defense-related genes *POD* and *CHI II* in response to the application of the seaweed extract was also reported. *POD* encodes the peroxidase enzyme, which has an antioxidant function against the reactive oxygen species (ROS) resulting from different stresses [41]. Triggering the ROS-scavenging system in pea plants due to the seaweed extract application was supported by a reduction in electrolyte leakage, and an increment in the activity of POD and PPO enzymes, reported in this study. In addition, the reported enhancing effect on the phenolic content in pea plants supports the non-enzymatic antioxidant mode of action. *CHI II* encodes the hydrolytic enzyme “chitinase”, which degrades the glycosidic bonds in the chitin molecule, the main component of the fungal cell wall [42]. This defense-related enzyme has an antifungal potential and is involved in the plant resistance responses against infection with phytopathogenic fungi [43]. The overexpression of these defense-related genes, reported in this study in response to treatment with the seaweed extract, confirms their contribution in the resistance-inducing effect of the seaweed extract. This result is in accordance with that obtained by Ali et al. [44], who found that treating tomato and sweet pepper plants with *A. nodosum* extract induced the plant resistance against *Xanthomonas campestris* pv. *vesicatoria* and *Alternaria solani*, and increased the activity of POD, PPO, chitinase, and *β*-1,3-glucanase enzymes. In addition, it upregulated the expression of different defense-related genes. An analysis of the *A. nodosum* extract showed that it contains abscisic acid and ethylene, which are multifunctional phytohormones, and are involved in various processes including growth, development, and responses against different biotic and abiotic stresses [14]. Both hormones are known as key signaling molecules, which regulate many stress-responsive genes via abscisic acid and ethylene-signaling pathways, inducing the plant resistance via variable mechanisms [45,46]. A transcriptomic analysis from previous studies indicated that the exogenous application of these phytohormones induced the endogenous signaling pathways in the plant, modulating their defense responses against pathogenic fungi [47,48]. The resistance-inducing effect of the seaweed extract can be attributed to these two constituents.

The results obtained from this study revealed that the mycorrhization of pea roots induced the plant resistance against *R. solani* infection, upregulated the tested defense-related genes (*JERF3*, *POD*, and *CHI II*), enhanced their phenolic content, and improved the activity of the antioxidant enzymes POD and PPO. These results are in agreement with the findings of EL-Sharkawy et al. [16], who found that the colonization of wheat plants with AMF induced the defense responses against stem rust disease. The application of AMF as biocontrol agents against different fungal, bacterial, and viral plant diseases has been extensively studied by many researchers [13,18]. The mycorrhization of the plant root leads to extensive genetic reprogramming in the plant cell, resulting in a set of physiological modulations that regulate the plant responses to different biotic and abiotic stresses [49]. Among these induced physiological modulations, the activation of multiple innate and adaptive immune responses takes place in the plant in order to defend itself against invading pathogens. However, the intensity of these modifications varies depending on the plant and fungus species, level of colonization, and the environmental conditions [50]. In this regard, the different modes of action have been discussed. Rashad et al. [17] found that most of the polyphenol biosynthetic pathway genes were overexpressed in sunflower roots due to their colonization with *R. irregularis*; higher due to infection with *R. solani*. Moreover, it was found that cell wall lignification and the production of fungitoxic polyphenolic substances were the main induced responses. This result is in agreement with the cell wall thickening observed during the TEM examination in this study. The activation of the hypersensitivity responses such as cytoplasmic granulation and programmed cell death was also reported in mycorrhizal common bean roots infected with *R. solani* [20]. The accumulation of the antioxidant enzymes and defense-related proteins represents another defensive mechanism, which has been reported in different plants in response to mycorrhizal colonization [13]. Increments in the activity of POD and PPO enzymes reported in this study are in accordance with this information. All of these defensive mechanisms concurrently work to inhibit the pathogen growth and restrict their invasion and transmission from cell to cell. However, the results obtained in this study revealed that the inducing effect of the dual treatment of the mycorrhizal-infected pea plants, treated with the seaweed extract at 3%, was higher than that of the single treatments. This indicates the synergistic effect of both mycorrhization and the seaweed extract treatment in triggering the plant resistance against the infection with *R. solani*, despite the decrease in the colonization intensity observed in the mycorrhizal pea roots, treated with the seaweed extract at 3%, compared with the mycorrhizal plants not treated with this extract.

One of the most interesting results reported in this study is the growth-promoting effect of the seaweed extract. This result is in agreement with the findings of Verma et al. [51], who reported the growth-stimulating effect of *A. nodosum* extract on *Vigna aconitifolia* plants, when applied as spray or root drench at 0.1%. The application of *A. nodosum* extract has repeatedly demonstrated their enhancing effect on the vegetative growth and yield of different plant species via various mechanisms [39]. One of the important growth-promoting mechanisms, reported for *A. nodosum* extract, is their constitution of multiple phytohormones [52]. In this regard, the analysis of the seaweed extract applied in this study showed that it contained different plant hormones, namely indole-3-acetic acid (IAA), cytokinin, abscisic acid, gibberellic acid, and ethylene. These plant hormones and their interactions have critical roles in different physiological and developmental processes, such as cell proliferation and elongation in the plant, by regulating the expression of many growth-responsive genes through crosstalk between different signaling pathways [53]. In addition, *A. nodosum* extract has been reported to enhance the growth of many plants by overexpressing the phytohormones’ biosynthesis-related genes. In this regard, Goñi et al. [54], reported the upregulation of the gibberellic acid biosynthesis-related genes (*GASA1* and *GASA4*) in Arabidopsis leaves when foliar sprayed with *A. nodosum* extract at 0.2%. Enhancing nutrient availability and uptake is another growth-promoting mechanism, which has been reported for the seaweed extract, and leads to the improvement of the overall vigor of the plant [55]. An analysis of the seaweed extract applied in this study showed that it contains an array of macro and micronutrients, which makes it a high potential bio-fertilizer. Ertani et al. [56] reported an enhancement in the nutrient contents of zinc, iron, boron, copper, magnesium, calcium, and manganese in maize leaves in response to the application of *A. nodosum* extract. The chemical characterization of five extracts from *A. nodosum* (different commercial products) was carried out by Ertani et al. [56] using Fourier transform infrared (FT-IR) and FT-Raman spectroscopies. High contents of IAA up to 32.4 nM, and phenolic compounds up to 1.9 mg L^−1^ were detected. In addition, significant contents of isopentenyladenosine, carbon, and nitrogen were also detected. However, the growth-inducing effect on maize plants and their physiological responses varied from one extract to another, depending on the chemical properties of each extract.

The colonization of plant roots with AMF has been extensively reported to enhance the growth and productivity of many crops via different growth-promoting mechanisms [57,58]. The stimulation of water and nutrient uptake in plants is one of the key benefits of the fungal partner during mycorrhizal symbiosis [59]. The extraradical hyphae of the mycorrhizal fungus extend in the soil, providing a better acquisition of water and nutrients for the host plant. Interestingly, these extraradical hyphae extend to colonize the roots of neighboring plants forming common mycorrhizal networks, which improve the capture and transfer of the nutrients to the neighboring plants more than the host plant [60]. In this regard, AMF have been reported to secrete some organic acids that increase the phyto-availability of many macro- and micro-nutrients in the soil; in particular, acid phosphatase, which is secreted under low phosphorous conditions [61]. Moreover, mycorrhizal colonization has been found to enhance the photosynthetic pigments, photosynthesis rate, and activity of various metabolic enzymes, and upregulate the expression of many growth-related genes [62]. The production of different phytohormones by AMF during mycorrhizal symbiosis has also been reported. Pons et al. [63] reported the production of isopentenyl adenosine, indole-3-acetic acid, gibberellin A4, and ethylene by *R. irregularis*. These phytohormones play vital roles in different metabolic and developmental processes in the host plant. In this study, the results showed that colonization with AMF and the seaweed extract application synergistically enhanced the growth of pea plants, and improved their photosynthetic pigments content.

## 5. Conclusions

Results from this study indicated the synergistic inducing effect of mycorrhizal colonization and treatment with the seaweed extract at 3% on upregulation of the defense-related genes (*JERF3*, *POD*, and *CHI* *II*). Moreover, other resistance plant responses were highly triggered, such as the antioxidant enzymes POD and PPO, and the accumulation of the phenolic compounds. At the ultrastructural level, various hypersensitivity alterations were induced in the plant cell, which led to the reduction of the disease severity. In addition, this dual treatment showed a synergistic growth-promoting effect on the pea plants and the enhanced content of the photosynthetic pigments in their leaves. Based on these results, it can be concluded that this dual treatment has a promising biocontrol activity against *R. solani*, and a growth-promoting effect on pea plants. However, the field application of this dual treatment should be evaluated prior to any use recommendations.

## Figures and Tables

**Table 1:** Product analysis of *Ascophyllum nodosum* Seaweed Extract.

Items	Results
Water-soluble	100%
Moisture	3%
pH	8
Alginic acid	18.9%
Organic matter	50%
Nitrogen (N)	1.8%
Phosphorus pentoxide (P_2_O_5_)	2.4%
Potassium oxide (K_2_O)	18.7%
Betaine	62 ppm
Cytokinin	200 ppm
Indole-3-acetic acid (IAA)	50 ppm
Gibberellin	18 ppm
Abscisic acid (ABA)	20 ppm
Ethylene	9 ppm
Polyamine	10 ppm
Amino Acid	2%
Mannitol	4%
Sulfur (S)	1%
Calcium (Ca)	1%
Magnesium (Mg)	0.2%
Sodium (Na)	1%
Boron (B)	0.2%
Iron (Fe)	200 ppm
Manganese (Mn)	2 ppm
Zinc (Zn)	27 ppm
Copper (Cu)	7 ppm

**Table 2:** Primer sequences of the investigated genes.

Gene Description	Abbrev.	Accession No.	Sequence (5′-3′)
Jasmonate and ethylene-responsive factor 3	*JERF3*-F *JERF3*-R	AY383630	GCCATTTGCCTTCTCTGCTTC GCAGCAGCATCCTTGTCTGA
Chitinase class II	*CHI ΙΙ*-F *CHI ΙΙ*-R	U30465	GCGTTGTGGTTCTGGATGACA CAGCGGCAGAATCAGCAACA
Peroxidase	*POD*-F *POD*-R	X94943	CCTTGTTGGTGGGCACACAA GGCCACCAGTGGAGTTGAAA
Elongation factor 1-α	*EF1-α*-F *EF1-α*-R	EC959059	GAACTGGGTGCTTGATAGGC
			AACCAAAATATCCGGAGTAAAAGA

**Table 3:** Mean growth parameters of pea plants (cv. Master-B) infected with Rhizoctonia root rot in response to treatment with the seaweed extract and/or colonization with arbuscular mycorrhizal fungi at 20 days post-inoculation *.

Treatment	Shoot Height (cm)	Root Length (cm)	Shoot Dry Weight (g)	Root Dry Weight (g)	Number of Leaves/Plant	Leaf Area (cm^2^)
C	45.0 ± 3.6 ^d^	5.3 ± 0.6 ^ef^	1.1 ± 0.3 ^e^	0.12 ± 0.02 ^e^	5.3 ± 1.0 ^cd^	37.7 ± 3.4 ^cd^
P	34.3 ± 4.2 ^e^	2.7 ± 0.3 ^g^	0.7 ± 0.1 ^f^	0.08 ± 0.01 ^f^	4.0 ± 0.5 ^d^	31.5 ± 2.5 ^f^
CM	50.7 ± 4.6 ^bc^	13.2 ± 0.9 ^a^	2.5 ± 0.4 ^a^	0.20 ± 0.03 ^b^	10.0 ± 1.2 ^a^	55.1 ± 4.3 ^a^
SW1	47.0 ± 3.5 ^cd^	6.3 ± 0.6 ^de^	1.6 ± 0.2 ^d^	0.11 ± 0.05 ^e^	6.3 ± 0.5 ^bcd^	39.8 ± 3.6 ^c^
SW2	47.7 ± 2.7 ^bc^	7.3 ± 0.4 ^cd^	1.8 ± 0.3 ^bcd^	0.16 ± 0.04 ^cd^	6.7 ± 0.4 ^bcd^	44.7 ± 3.8 ^b^
SW3	49.0 ± 3.1 ^bc^	7.7 ± 0.7 ^cd^	2.2 ± 0.4 ^bc^	0.17 ± 0.03 ^c^	7.7 ± 0.6 ^abcd^	46.6 ± 3.4 ^b^
PF	42.3 ± 3.6 ^d^	4.7 ± 0.6 ^fg^	1.0 ± 0.4 ^e^	0.10 ± 0.04 ^ef^	5.3 ± 0.6 ^cd^	34.7 ± 2.7 ^de^
PM	49.0 ± 2.6 ^bc^	9.1 ± 0.4 ^bcd^	2.2 ± 0.6 ^bc^	0.19 ± 0.08 ^bc^	8.7 ± 0.7 ^abc^	36.2 ± 2.6 ^d^
PFM	47.3 ± 3.3 ^cd^	7.4 ± 0.3 ^cde^	1.6 ± 0.4 ^d^	0.13 ± 0.03 ^de^	6.3 ± 0.7 ^bcd^	35.3 ± 2.6 ^de^
SW1M	47.0 ± 4.1 ^cd^	8.0 ± 0.4 ^cd^	1.8 ± 0.3 ^bcd^	0.19 ± 0.04 ^bc^	8.3 ± 1.1 ^abc^	37.9 ± 2.7 ^cd^
SW2M	49.3 ± 3.7 ^bc^	9.0 ± 0.5 ^bcd^	2.3 ± 0.4 ^ab^	0.20 ± 0.05 ^b^	8.7 ± 1.2 ^abc^	44.7 ± 3.4 ^b^
SW3M	57.7 ± 4.2 ^a^	11.7 ± 1.0 ^ab^	2.5 ± 0.8 ^a^	0.24 ± 0.06 ^a^	9.7 ± 1.3 ^ab^	57.1 ± 2.9 ^a^
PSW1	48.0 ± 2.8 ^bc^	5.0 ± 0.8 ^ef^	1.5 ± 0.6 ^d^	0.13 ± 0.04 ^de^	6.0 ± 0.6 ^bcd^	32.1 ± 2.8 ^f^
PSW2	52.3 ± 3.7 ^bc^	5.3 ± 0.8 ^ef^	1.9 ± 0.5 ^bc^	0.14 ± 0.03 ^de^	6.3 ± 0.7 ^bcd^	36.7 ± 2.6 ^d^
PSW3	55.0 ± 4.8 ^ab^	6.7 ± 0.5 ^de^	2.0 ± 0.4 ^b^	0.16 ± 0.05 ^cd^	7.3 ± 0.4 ^abcd^	39.6 ± 3.4 ^c^
PSW1M	49.3 ± 4.6 ^bc^	7.0 ± 0.4 ^de^	1.6 ± 0.5 ^d^	0.16 ± 0.04 ^cd^	7.3 ± 0.6 ^abcd^	33.6 ± 3.3 ^ef^
PSW2M	54.3 ± 3.4 ^ab^	7.7 ± 0.6 ^cde^	2.1 ± 0.6 ^bc^	0.17 ± 0.04 ^c^	7.7 ± 0.5 ^abcd^	36.9 ± 3.6 ^d^
PSW3M	55.7 ± 3.8 ^ab^	10.0 ± 0.4 ^bc^	2.2 ± 0.6 ^bc^	0.18 ± 0.06 ^bc^	8.7 ± 0.8 ^abc^	40.8 ± 4.0 ^c^

* In each column, values followed by the same letter are not significantly different according to Tukey’s HSD test (*p* ≤ 0.05), each value represents the mean of ten replicates ± SD. Where, C: uninfected and non-mycorrhizal, CM: uninfected and mycorrhizal, P: infected and non-mycorrhizal, PM: infected and mycorrhizal, PF: infected, non-mycorrhizal and treated with fungicide (Tendro 40% FS), PFM: infected, mycorrhizal, and treated with fungicide, SW1, SW2, and SW3: uninfected, non-mycorrhizal, and treated with the seaweed extract at 1, 2, and 3%, respectively, SW1M, SW2M, and SW3M: uninfected, mycorrhizal, and treated with the seaweed extract at 1, 2, and 3%, respectively, PSW1, PSW2, and PSW3: infected, non-mycorrhizal, and treated with the seaweed extract at 1, 2, and 3%, respectively, PSW1M. PSW2M, and PSW3M: infected, mycorrhizal, and treated with the seaweed extract at 1, 2, and 3%, respectively.

**Table 4:** Mean yield and its components of pea plants (cv. Master-B) infected with Rhizoctonia root rot in response to treatment with the seaweed extract and/or colonization with arbuscular mycorrhizal fungi, at 40 days post-inoculation *.

Treatment	No. of Pods/Plant	Pod Weight (g)	Pod Length (cm)	Pod Width (cm)	Yield/Plant (g)	No. of Seeds/Pod
C	2.0 ± 0.8 ^c^	3.0 ± 0.2 ^cd^	5.2 ± 0.3 ^bc^	1.3 ± 0.10 ^b^	6.0 ± 1.0 ^h^	3.3 ± 0.6 ^bc^
P	1.3 ± 0.6 ^d^	2.1 ± 0.3 ^e^	4.5 ± 0.8 ^c^	1.1 ± 0.06 ^c^	3.1 ± 1.4 ^i^	2.3 ± 0.5 ^c^
CM	3.3 ± 0.5 ^ab^	4.8 ± 0.6 ^ab^	6.2 ± 0.3 ^ab^	1.4 ± 0.07 ^ab^	16.1 ± 3.5 ^b^	5.7 ± 0.7 ^ab^
SW1	2.3 ± 0.5 ^bc^	4.2 ± 0.5 ^b^	6.1 ± 0.4 ^ab^	1.4 ± 0.04 ^ab^	9.5 ± 1.1 ^fg^	4.0 ± 0.9 ^abc^
SW2	2.7 ± 0.6 ^bc^	4.2 ± 0.4 ^b^	6.3 ± 0.6 ^ab^	1.4 ± 0.05 ^ab^	11.3 ± 1.3 ^f^	5.0 ± 0.8 ^ab^
SW3	3.0 ± 0.9 ^ab^	5.1 ± 0.5 ^a^	6.5 ± 0.4 ^a^	1.4 ± 0.06 ^ab^	15.4 ± 1.5 ^cd^	5.7 ± 0.7 ^ab^
PF	2.0 ± 0.7 ^c^	2.9 ± 0.2 ^d^	5.1 ± 0.5 ^bc^	1.3 ± 0.07 ^b^	5.8 ± 0.9 ^h^	3.3 ± 0.5 ^bc^
PM	3.0 ± 0.4 ^ab^	4.7 ± 0.6 ^ab^	5.9 ± 0.7 ^ab^	1.4 ± 0.06 ^ab^	13.8 ± 1.5 ^d^	5.0 ± 0.6 ^ab^
PFM	3.0 ± 0.6 ^ab^	3.6 ± 0.3 ^c^	6.2 ± 0.6 ^ab^	1.4 ± 0.06 ^ab^	10.8 ± 1.0 ^f^	5.3 ± 0.6 ^ab^
SW1M	3.3 ± 0.7 ^ab^	4.4 ± 0.4 ^ab^	6.3 ± 0.5 ^ab^	1.4 ± 0.05 ^ab^	15.3 ± 2.1 ^cd^	4.7 ± 0.5 ^abc^
SW2M	3.9 ± 0.5 ^a^	4.6 ± 0.4 ^ab^	6.5 ± 0.7 ^a^	1.5 ± 0.09 ^a^	16.4 ± 2.2 ^b^	5.3 ± 0.4 ^ab^
SW3M	4.3 ± 0.9 ^a^	5.5 ± 0.8 ^a^	6.8 ± 0.8 ^a^	1.5 ± 0.07 ^a^	24.0 ± 3.6 ^a^	6.0 ± 0.5 ^a^
PSW1	2.3 ± 0.5 ^bc^	3.9 ± 0.8 ^bc^	6.1 ± 0.7 ^ab^	1.4 ± 0.08 ^ab^	8.7 ± 1.2 ^g^	5.0 ± 0.7 ^ab^
PSW2	3.7 ± 0.4 ^ab^	4.2 ± 0.6 ^b^	6.2 ± 0.4 ^ab^	1.4 ± 0.05 ^ab^	15.6 ± 1.7 ^bc^	5.3 ± 0.6 ^ab^
PSW3	3.3 ± 0.8 ^ab^	4.6 ± 0.4 ^ab^	6.3 ± 0.5 ^ab^	1.4 ± 0.04 ^ab^	15.7 ± 1.4 ^bc^	5.7 ± 0.5 ^ab^
PSW1M	3.0 ± 0.6 ^ab^	4.1 ± 0.3 ^b^	6.0 ± 0.4 ^ab^	1.4 ± 0.06 ^ab^	12.3 ± 1.3 ^e^	5.0 ± 0.6 ^ab^
PSW2M	3.0 ± 0.4 ^ab^	4.6 ± 0.6 ^ab^	6.2 ± 0.6 ^ab^	1.5 ± 0.07 ^a^	13.8 ± 1.7 ^d^	6.0 ± 0.8 ^a^
PSW3M	3.7 ± 0.5 ^ab^	5.0 ± 0.8 ^a^	6.3 ± 0.5 ^ab^	1.5 ± 0.04 ^a^	18.4 ± 2.0 ^ab^	6.0 ± 0.7 ^a^

* In each column, values followed by the same letter are not significantly different according to Tukey’s HSD test (*p* ≤ 0.05), each value represents the mean of ten replicates ± SD. Where, C: uninfected and non-mycorrhizal, CM: uninfected and mycorrhizal, P: infected and non-mycorrhizal, PM: infected and mycorrhizal, PF: infected, non-mycorrhizal and treated with fungicide (Tendro 40% FS), PFM: infected, mycorrhizal, and treated with fungicide, SW1, SW2, and SW3: uninfected, non-mycorrhizal, and treated with the seaweed extract at 1, 2, and 3%, respectively, SW1M, SW2M, and SW3M: uninfected, mycorrhizal, and treated with the seaweed extract at 1, 2, and 3%, respectively, PSW1, PSW2, and PSW3: infected, non-mycorrhizal, and treated with the seaweed extract at 1, 2, and 3%, respectively, PSW1M. PSW2M, and PSW3M: infected, mycorrhizal, and treated with the seaweed extract at 1, 2, and 3%, respectively.

**Table 5:** Mean phenolic content, activity of antioxidant enzymes, electrolyte leakage, and total soluble solids of pea roots (cv. Master-B) infected with Rhizoctonia root rot in response to treatment with the seaweed extract and/or colonization with arbuscular mycorrhizal fungi at 20 days post-inoculation *.

Treatment	Phenolic Content (mg.g^−1^ fwt)	Peroxidase (∆A_470_ min^−1^ g^−1^ fwt)	Polyphenol Oxidase (∆A_420_ min^−1^ g^−1^ fwt)	Electrolyte Leakage (%)	Soluble Solids Content (°Brix)
C	394.6 ± 7.8 ^i^	1.02 ± 0.08 ^h^	1.03 ± 0.07 ^i^	55.3 ± 1.4 ^f^	15.3 ± 0.8 ^bc^
P	568.9 ± 10.1 ^gh^	1.41 ± 0.0.3 ^efg^	1.33 ± 0.06 ^gh^	118.4 ± 1.9 ^a^	10.0 ± 0.4 ^d^
CM	584.3 ± 6.5 ^fgh^	1.89 ± 0.06 ^d^	1.54 ± 0.07 ^def^	55.5 ± 1.4 ^f^	17.0 ± 0.7 ^ab^
SW1	583.6 ± 8.1 ^fgh^	1.31 ± 0.04 ^fg^	1.20 ± 0.04 ^hi^	55.7 ± 1.1 ^f^	16.5 ± 0.8 ^ab^
SW2	605.1 ± 11.2 ^fgh^	1.36 ± 0.05 ^fg^	1.24 ± 0.06 ^h^	55.9 ± 0.9 ^f^	16.7 ± 0.6 ^ab^
SW3	623.7 ± 9.6 ^fgh^	1.39 ± 0.05 ^efg^	1.28 ± 0.07 ^h^	55.9 ± 1.2 ^f^	18.6 ± 0.7 ^a^
PF	531.6 ± 7.4 ^h^	1.30 ± 0.07 ^g^	1.27 ± 0.05 ^h^	87.2 ± 1.4 ^cd^	13.1 ± 0.9 ^c^
PM	900.9 ± 12.3 ^cd^	2.04 ± 0.05 ^cd^	1.69 ± 0.07 ^bcd^	86.9 ± 1.4 ^cd^	16.8 ± 0.5 ^ab^
PFM	690.5 ± 8.9 ^ef^	1.98 ± 0.08 ^cd^	1.54 ± 0.09 ^def^	87.4 ± 0.9 ^cd^	16.7 ± 1.0 ^ab^
SW1M	642.4 ± 10.1 ^fgh^	1.42 ± 0.03 ^efg^	1.33 ± 0.04 ^gh^	55.8 ± 1.0 ^f^	17.2 ± 0.9 ^ab^
SW2M	657.8 ± 6.9 ^fg^	1.53 ± 0.07 ^ef^	1.35 ± 0.03 ^fgh^	55.9 ± 0.9 ^f^	17.7 ± 1.2 ^ab^
SW3M	677.8 ± 7.4 ^efg^	1.60 ± 0.08 ^e^	1.39 ± 0.04 ^efgh^	57.5 ± 1.2 ^f^	18.5 ± 1.6 ^a^
PSW1	796.7 ± 11.0 ^de^	1.96 ± 0.08 ^cd^	1.51 ± 0.07 ^defg^	97.5 ± 1.4 ^b^	14.6 ± 0.7 ^b^
PSW2	878.9 ± 11.8 ^cd^	1.96 ± 0.09 ^cd^	1.56 ± 0.05 ^de^	86.5 ± 1.1 ^cd^	15.9 ± 1.0 ^abc^
PSW3	945.6 ± 10.5 ^c^	2.13 ± 0.06 ^abc^	1.64 ± 0.06 ^cd^	85.5 ± 1.2 ^d^	15.9 ± 0.4 ^abc^
PSW1M	1091.2 ± 18.8 ^b^	2.12 ± 0.07 ^bcd^	1.80 ± 0.07 ^abc^	89.7 ± 1.3 ^c^	16.5 ± 0.8 ^ab^
PSW2M	1158.7 ± 20.2 ^b^	2.32 ± 0.06 ^ab^	1.86 ± 0.08 ^ab^	85.4 ± 1.4 ^d^	17.1 ± 0.7 ^ab^
PSW3M	1346.4 ± 23.6 ^a^	2.36 ± 0.07 ^a^	1.97 ± 0.06 ^a^	78.1 ± 1.0 ^e^	17.4 ± 1.0 ^ab^

* In each column, values followed by the same letter are not significantly different according to Tukey’s HSD test (*p* ≤ 0.05), each value represents the mean of ten replicates ± SD. Where, C: uninfected and non-mycorrhizal, CM: uninfected and mycorrhizal, P: infected and non-mycorrhizal, PM: infected and mycorrhizal, PF: infected, non-mycorrhizal and treated with fungicide (Tendro 40% FS), PFM: infected, mycorrhizal, and treated with fungicide, SW1, SW2, and SW3: uninfected, non-mycorrhizal, and treated with the seaweed extract at 1, 2, and 3%, respectively, SW1M, SW2M, and SW3M: uninfected, mycorrhizal, and treated with the seaweed extract at 1, 2, and 3%, respectively, PSW1, PSW2, and PSW3: infected, non-mycorrhizal, and treated with the seaweed extract at 1, 2, and 3%, respectively, PSW1M. PSW2M, and PSW3M: infected, mycorrhizal, and treated with the seaweed extract at 1, 2, and 3%, respectively.

**Table 6:** Effects of application of the seaweed extract and/or colonization with arbuscular mycorrhizal fungi on total photosynthetic pigments in leaves of pea infected with Rhizoctonia root rot at 20 dpi *.

Treatment	Chl. *a* (mg g^−1^ fwt)	Chl. *b* (mg g^−1^ fwt)	Carotenoids (mg g^−1^ fwt)	Total Pigments (mg g^−1^ fwt)
C	2.34 ± 0.4 ^cd^	0.71 ± 0.08 ^ab^	0.50 ± 0.06 ^a^	3.55 ± 0.9 ^cde^
P	0.62 ± 0.2 ^f^	0.24 ± 0.03 ^bc^	0.02 ± 0.004 ^b^	0.88 ± 0.03 ^h^
CM	2.60 ± 0.7 ^bc^	1.02 ± 0.04 ^a^	0.51 ± 0.04 ^a^	4.13 ± 0.8 ^b^
SW1	2.34 ± 0.6 ^cd^	0.75 ± 0.03 ^ab^	0.43 ± 0.03 ^ab^	3.52 ± 0.6 ^cde^
SW2	2.72 ± 0.5 ^bc^	0.84 ± 0.04 ^ab^	0.42 ± 0.04 ^ab^	3.98 ± 0.6 ^bc^
SW3	3.44 ± 0.7 ^a^	0.93 ± 0.06 ^ab^	0.61 ± 0.05 ^a^	4.98 ± 0.5 ^a^
PF	1.75 ± 0.4 ^de^	0.71 ± 0.04 ^ab^	0.33 ± 0.05 ^ab^	2.79 ± 0.7 ^ef^
PM	2.01 ± 0.6 ^d^	0.55 ± 0.04 ^bc^	0.42 ± 0.03 ^ab^	2.98 ± 0.8 ^e^
PFM	2.22 ± 0.6 ^cd^	0.54 ± 0.04 ^bc^	0.44 ± 0.04 ^ab^	3.20 ± 0.4 ^de^
SW1M	2.61 ± 0.5 ^bc^	0.84 ± 0.06 ^ab^	0.25 ± 0.02 ^ab^	3.70 ± 0.5 ^cd^
SW2M	2.90 ± 0.5 ^ab^	0.86 ± 0.05 ^ab^	0.36 ± 0.04 ^ab^	4.12 ± 0.8 ^bc^
SW3M	3.66 ± 0.4 ^a^	1.12 ± 0.06 ^a^	0.61 ± 0.03 ^a^	5.39 ± 1.0 ^a^
PSW1	1.14 ± 0.6 ^e^	0.53 ± 0.05 ^bc^	0.20 ± 0.04 ^ab^	1.87 ± 0.3 ^g^
PSW2	1.23 ± 0.4 ^e^	0.53 ± 0.06 ^bc^	0.24 ± 0.03 ^ab^	2.00 ± 0.4 ^g^
PSW3	2.33 ± 0.9 ^cd^	0.64 ± 0.04 ^b^	0.43 ± 0.04 ^ab^	3.40 ± 0.6 ^cde^
PSW1M	2.35 ± 0.6 ^cd^	0.55 ± 0.07 ^bc^	0.26 ± 0.03 ^ab^	3.16 ± 0.5 ^e^
PSW2M	2.72 ± 0.9 ^bc^	0.63 ± 0.04 ^b^	0.25 ± 0.04 ^ab^	3.60 ± 0.8 ^cde^
PSW3M	2.74 ± 0.8 ^bc^	0.74 ± 0.08 ^ab^	0.33 ± 0.05 ^ab^	3.81 ± 0.6 ^bcd^

* In each column, values followed by the same letter are not significantly different according to Tukey’s HSD test (*p* ≤ 0.05); each value represents the mean of three replicates ± SD. Where, C: uninfected and non-mycorrhizal, CM: uninfected and mycorrhizal, P: infected and non-mycorrhizal, PM: infected and mycorrhizal, PF: infected, non-mycorrhizal and treated with fungicide (Tendro 40% FS), PFM: infected, mycorrhizal, and treated with fungicide, SW1, SW2, and SW3: uninfected, non-mycorrhizal, and treated with seaweed extract at 1, 2, and 3%, respectively, SW1M, SW2M, and SW3M: uninfected, mycorrhizal, and treated with seaweed extract at 1, 2, and 3%, respectively, PSW1, PSW2, and PSW3: infected, non-mycorrhizal, and treated with seaweed extract at 1, 2, and 3%, respectively, PSW1M. PSW2M, and PSW3M: infected, mycorrhizal, and treated with seaweed extract at 1, 2, and 3%, respectively.

**Table 7:** Mycorrhizal colonization in roots of pea (cv. Master-B) infected with Rhizoctonia root rot and/or treated with the seaweed extract at 30 days post-inoculation *.

Treatment	FC (%)	IC (%)	FA (%)
C	0 ^d^	0 ^f^	0 ^e^
P	0 ^d^	0 ^f^	0 ^e^
CM	88.3 ± 3.7 ^a^	50.7 ± 2.4 ^a^	20.6 ± 1.4 ^a^
SW1	0 ^d^	0 ^f^	0 ^e^
SW2	0 ^d^	0 ^f^	0 ^e^
SW3	0 ^d^	0 ^f^	0 ^e^
PF	0 ^d^	0 ^f^	0 ^e^
PM	69.4 ± 1.1 ^b^	34.5 ± 1.6 ^c^	12.4 ± 0.9 ^cd^
PFM	52.1 ± 0.6 ^c^	26.4 ± 1.2 ^e^	9.7 ± 0.4 ^d^
SW1M	85.6 ± 2.5 ^a^	46.4 ± 1.8 ^ab^	18.7 ± 0.9 ^ab^
SW2M	83.9 ± 1.8 ^a^	40.9 ± 2.1 ^b^	17.9 ± 1.0 ^ab^
SW3M	86.4 ± 1.1 ^a^	44.5 ± 0.9 ^b^	18.4 ± 0.8 ^ab^
PSW1	0 ^d^	0 ^f^	0 ^e^
PSW2	0 ^d^	0 ^f^	0 ^e^
PSW3	0 ^d^	0 ^f^	0 ^e^
PSW1M	70.2 ± 0.8 ^b^	30.7 ± 0.9 ^d^	11.8 ± 0.8 ^cd^
PSW2M	72.8 ± 1.0 ^b^	33.4 ± 1.1 ^c^	12.0 ± 0.7 ^cd^
PSW3M	69.6 ± 0.9 ^b^	35.1 ± 1.0 ^c^	12.2 ± 0.5 ^cd^

* In each column, values followed by the same letter are not significantly different according to Tukey’s HSD test (*p* ≤ 0.05), each value represents the mean of three replicates ± SD. Where, FC = frequency of colonization, IC = intensity of colonization, and FA = frequency of arbuscules, C: uninfected and non-mycorrhizal, CM: uninfected and mycorrhizal, P: infected and non-mycorrhizal, PM: infected and mycorrhizal, PF: infected, non-mycorrhizal and treated with fungicide (Tendro 40% FS), PFM: infected, mycorrhizal, and treated with fungicide, SW1, SW2, and SW3: uninfected, non-mycorrhizal, and treated with seaweed extract at 1, 2, and 3%, respectively, SW1M, SW2M, and SW3M: uninfected, mycorrhizal, and treated with seaweed extract at 1, 2, and 3%, respectively, PSW1, PSW2, and PSW3: infected, non-mycorrhizal, and treated with seaweed extract at 1, 2, and 3%, respectively, PSW1M. PSW2M, and PSW3M: infected, mycorrhizal, and treated with seaweed extract at 1, 2, and 3%, respectively.

## Data Availability

Not applicable.
